# A Biomimetic Breast Tumor‐On‐Chip Platform Reveals Mesenchymal Marker Upregulation Following Gold Nanorod Photothermal Therapy

**DOI:** 10.1002/smll.75343

**Published:** 2026-08-24

**Authors:** Camila A. Salvego, Hasti Honari, Anastasiia Dubrova, Stéphanie Descroix, Martha S. Ribeiro, Aurore V. Walle, Claire Wilhelm

**Affiliations:** ^1^ Centro de Lasers e Aplicações Instituto de Pesquisas Energéticas e Nucleares (IPEN‐CNEN) São Paulo Brazil; ^2^ Laboratoire Physique des Cellules et Cancer PCC CNRS UMR168 Institut Curie Sorbonne Université PSL Research University Paris France

**Keywords:** hyperthermia, mesenchymal transition, microfluidics, near‐infrared laser, tumor microenvironment

## Abstract

Photothermal therapy (PTT) using plasmonic nanoparticles has emerged as a promising strategy for localized cancer treatment, yet physiologically relevant models for assessing cell‐line model‐dependent responses remain limited. Here, we engineered a breast tumor‐on‐chip platform to investigate gold nanorod (AuNR)‐mediated PTT in a 3D type I collagen microenvironment using luminal‐like MCF7 and triple‐negative breast cancer MDA‐MB‐231 spheroids. A custom microfluidic architecture incorporating sliding‐wall technology enabled controlled lateral perfusion with a spatially restricted thermal response consistent with localized AuNR‐mediated heating within the central matrix compartment. Both tumor models exhibited pronounced cytotoxicity at temperatures above ∼50°C and showed comparable half‐maximal responses in the Live/Dead ratio dose‐response curves, corresponding to laser powers of approximately 0.48 and 0.38 W for MCF7 and MDA‐MB‐231 spheroids, respectively, despite their distinct epithelial and mesenchymal phenotypes. Immunofluorescence analyses 24 h after sublethal PTT revealed protein‐level changes, including an increased vimentin‐to‐E‐cadherin ratio and enhanced N‐cadherin expression, particularly in MCF7 spheroids. Complementary gene expression analysis revealed treatment‐associated modulation of apoptosis‐, inflammation‐, and epithelial‐mesenchymal transition (EMT)‐associated genes, although most changes in EMT‐associated transcripts were modest and not statistically significant. Altogether, this work suggests treatment‐associated alterations in selected mesenchymal markers in MCF7 spheroids following sublethal photothermal stress, warranting further temporal and functional validation.

## Graphical abstract

A biomimetic microfluidic tumor‐on‐chip platform was designed to uncover subtype‐dependent responses to nanoparticle‐mediated photothermal therapy in breast cancer spheroids. The platform establishes a physiologically relevant and controllable 3D system for precision evaluation of nanophotothermal cancer therapies. Real‐time light‐triggered heating induced distinct structural and molecular alterations in luminal and triple‐negative models, highlighting the influence of tumor phenotype on therapeutic outcome.

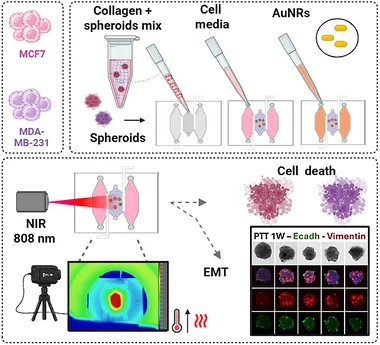

## 1 Introduction

Breast cancer (BC) remains the most frequently diagnosed malignancy among women globally and is a highly heterogeneous disease [1]. It continues to be a major contributor to cancer‐associated deaths, with an estimated 2.3 million incident cases and 666 000 deaths reported by the latest GLOBOCAN in 2022 [2]. Patient prognosis is largely determined by molecular subtype, disease stage at diagnosis, and response to therapy [3, 4].

Among the various BC subtypes, luminal tumors are generally associated with hormone receptor expression and more favorable clinical outcomes, whereas triple‐negative breast cancer (TNBC), which lacks estrogen receptor, progesterone receptor, and HER2 (Human Epidermal growth factor Receptor 2) expression, is typically linked to higher aggressiveness, increased metastatic potential, and limited targeted treatment options [5]. These subtype‐dependent differences highlight the biological complexity of BC and the need for experimental platforms capable of capturing heterogeneous tumor behaviors and therapy responses.

Photothermal therapy (PTT) has emerged as a promising approach for treating BC [6, 7, 8, 9]. By converting light into heat, PTT elevates cell temperature beyond physiological limits and may induce cellular damage through membrane disruption, protein denaturation, and subsequent cell death [10]. The effectiveness of PTT is strongly associated with the efficient accumulation and activation of photothermal agents within the tumor region, thereby enabling targeted thermal ablation of malignant tissue while reducing injury to adjacent healthy areas [11, 12]. Among the photothermal agents explored, gold nanoparticles (AuNPs) have shown significant promise for PTT applications due to their unique optical properties and biocompatibility [13]. Their size, shape, and surface chemistry can be tailored to enhance absorption in the near‐infrared (NIR) region [14, 15], where light penetrates more deeply and more effectively in tissues. This facilitates efficient, localized heating at the tumor site, enabling effective thermal destruction of cancer cells [16].

Despite its potential, the clinical translation of NP‐mediated PTT (NP‐PTT) still faces several challenges. One critical challenge is the tumor microenvironment (TME), a complex and dynamic milieu composed of diverse cell types and the extracellular matrix (ECM), which provides mechanical support to the tumor and mediates intercellular communication [17, 18]. In particular, TME may restrict NP perfusion and distribution, thereby influencing heat transfer and altering therapeutic efficacy [18]. Additionally, the intricate network of ECM components, including stromal cells and biochemical gradients, not only affects treatment outcomes but also plays a crucial role in tumor progression and therapy resistance in BC [17, 19]. These microenvironmental constituents participate in several biological programs that contribute to tumor plasticity, including epithelial‐mesenchymal transition (EMT). During EMT, epithelial cells progressively downregulate epithelial features, including cell‐cell adhesion mechanisms and markers such as E‐cadherin, while acquiring a mesenchymal‐like phenotype characterized, for instance, by increased vimentin expression. This phenotypic conversion is associated with enhanced migratory capacity, thereby facilitating local invasion and metastatic dissemination [20]. Although EMT has been strongly linked to highly aggressive BC phenotypes, including TNBC, this process is not restricted to this subtype [20, 21, 22].

3D culture platforms have emerged as more physiologically relevant for reproducing key features of the TME, including multicellular architecture, spatial organization, and cell‐matrix interactions [23, 24, 25, 26]. By preserving critical biophysical and biochemical cues, these systems enable improved investigation of tumor progression and therapeutic responses. However, many conventional 3D models still incompletely recapitulate essential components of the TME, particularly ECM organization and dynamic transport conditions, which can strongly influence therapeutic responses [27].

To address these challenges, tumor‐on‐a‐chip (ToC) platforms have emerged as a promising alternative. These microfluidic systems integrate 3D cultures within precisely controlled microenvironments that more closely replicate in vivo conditions [28, 29, 30]. The ToC approach offers a reproducible, human‐relevant platform for evaluating NP‐mediated PTT, enabling precise control of key parameters such as NP concentration, laser exposure, and temperature. Furthermore, by enabling fine‐tuned regulation of the ECM's physical and biochemical properties, the ToC system provides a powerful tool for investigating localized thermal responses and NP‐PTT‐induced effects at high resolution.

Despite growing interest in NP‐PTT, few studies have investigated these approaches in ToC systems [31, 32], and, to the best of our knowledge, none have compared thermally induced responses across two biologically distinct BC cell‐line models, including a TNBC cell‐line model. Importantly, the novelty of this study does not rely solely on AuNR‐mediated PTT but rather on its integration into a controllable, biomimetic ToC platform that enables evaluation of cell‐line model‐dependent responses under spatially defined photothermal conditions. Here, we engineered a breast ToC platform incorporating sliding‐wall technology [33] to investigate responses to NP‐PTT in biologically distinct BC models. MCF7 and MDA‐MB‐231 cell lines were selected as representative models of luminal‐like and TNBC, respectively, owing to their distinct molecular characteristics and epithelial‐mesenchymal phenotypes. MCF7 cells display a predominantly epithelial phenotype, characterized by high E‐cadherin expression and limited invasive potential, whereas MDA‐MB‐231 cells exhibit a mesenchymal‐like phenotype, with high expression of N‐cadherin and vimentin, reduced E‐cadherin expression, and enhanced migratory and invasive capacities [34]. Spheroids from both cell lines were embedded in a type I collagen matrix within a custom microfluidic device incorporating sliding‐wall technology to reproduce key features of the TME. Beyond evaluating photothermal cytotoxicity, we investigated apoptosis‐ and inflammation‐associated molecular responses, as well as sublethal PTT‐induced modulation of epithelial and mesenchymal markers.

## 2 Results

### 2.1 Spheroid Formation and Microfluidic Chip Development

Figure 1 presents a schematic timeline outlining the fabrication of the ToC platform incorporating BC spheroids embedded in a collagen type I matrix. Briefly, MCF7 and MDA‐MB‐231 spheroids were formed in agarose microwells, mixed with a 4 mg·ml^−1^ collagen I prepolymer solution (Figure 1A), and introduced into a microfluidic chip with sliding walls (Figure 1B,C). For spheroid formation, custom molds compatible with a 6‐well plate format were used. Each mold, designed to fit a single well, contained 2200 micropillars (200 µm in height and 200 µm in diameter), fabricated by 3D printing.

**FIGURE 1:**
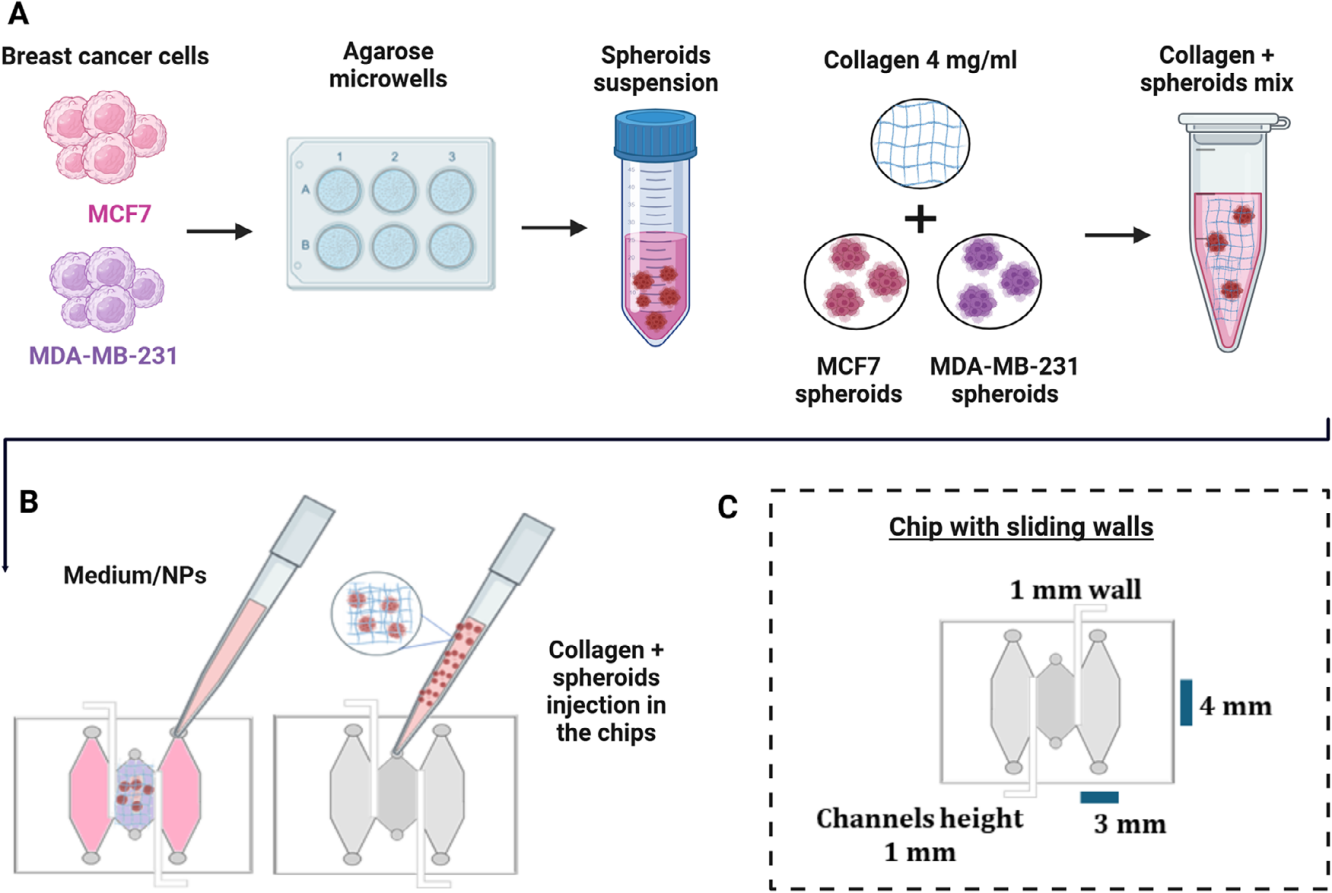
Spheroid formation and integration into microfluidic chips. (A) Schematic of the workflow for generating MCF7 and MDA‐MB‐231 spheroids using agarose microwell molds in a standard 6‐well plate, where 0.5 million cells were seeded and allowed to self‐assemble into spheroids. Formed spheroids were harvested via gentle pipetting, centrifuged to remove excess medium, and resuspended in a collagen I solution (4 mg mL^−^^1^). (B) The spheroid‐collagen mixture was then injected into the central channel of the microfluidic chip. After collagen polymerization, culture medium with/wo AuNRs was added to the lateral channels. (C) Microfluidic device incorporating sliding walls. Created with BioRender.

Cells were plated at densities between 125 and 1000 cells per microwell and allowed to self‐assemble into spheroids (Figure 2A,B for MCF7 and MDA‐MB‐231, respectively). MCF7 cells, used here as a luminal‐like BC cell‐line model and characterized by epithelial‐like features, formed densely packed spheroids with strong intercellular cohesion and clear edges within the agarose microwells in just 24 h. The ability of these cells to rapidly organize into uniform spheroids is consistent with the pronounced intercellular cohesion and epithelial‐like phenotype of MCF7 cells. By contrast, MDA‐MB‐231 cells, used here as a TNBC cell‐line model, required a longer culture period to form spheroidal structures. Even after prolonged incubation, the resulting spheroids exhibited a loose architecture and poorly defined borders, consistent with the mesenchymal phenotype and reduced intercellular cohesion characteristic of MDA‐MB‐231 cells. We analyzed and quantified spheroid morphology and growth dynamics, with quantitative area‐based growth curves shown in Figure 2C,D. For both cell lines, spheroid area was influenced by culture time and the initial cell seeding density.

**FIGURE 2:**
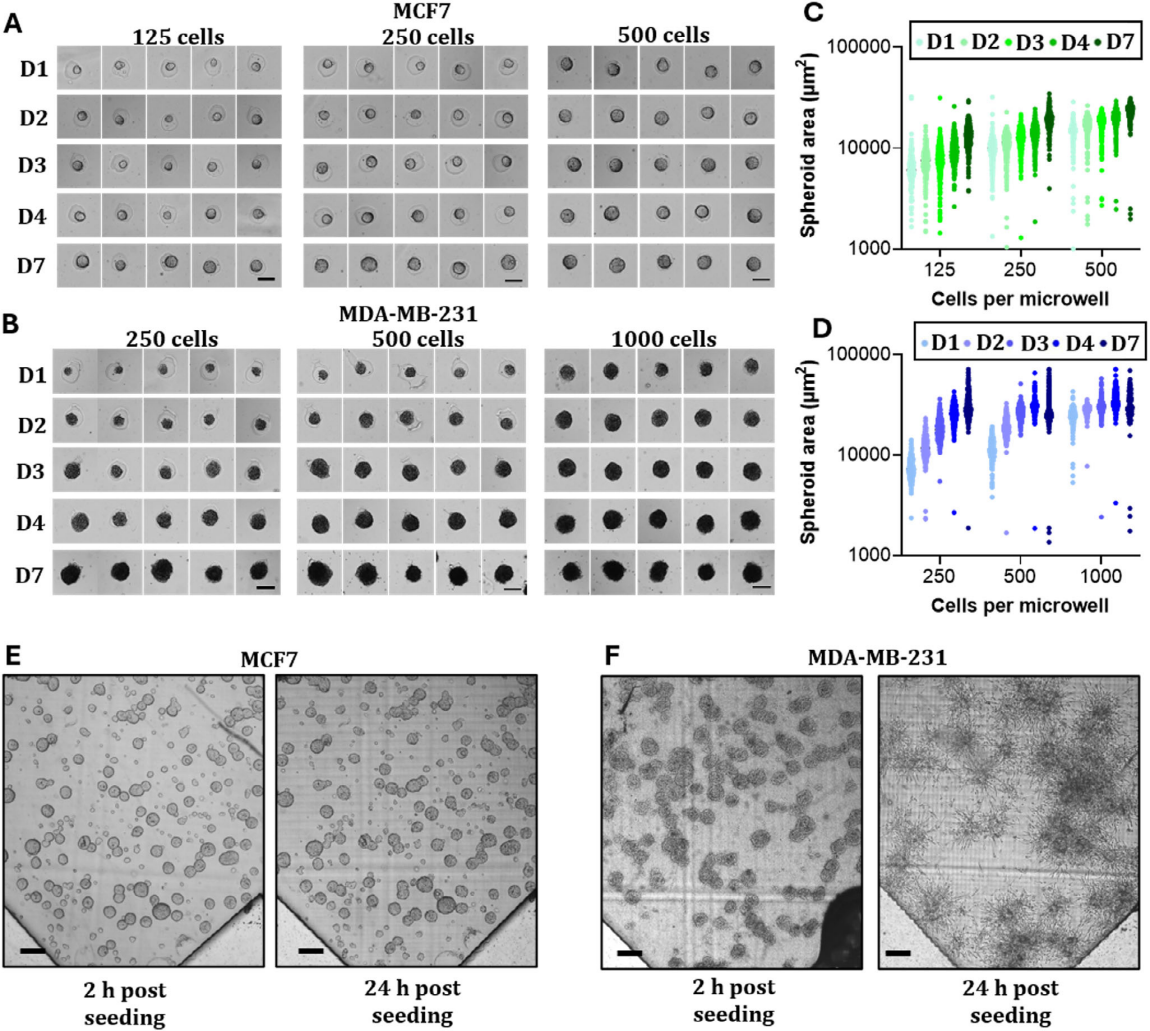
Characterization of BC spheroids before and after microfluidic chip loading. (A‐D) Characterization of spheroid morphology and growth dynamics within the microwells prior to chip injection. Cells were seeded at three different densities: 125, 250, and 500 cells per microwell for MCF7, and 250, 500, and 1000 cells per microwell for MDA‐MB‐231. Representative brightfield images of MCF7 (A) and MDA‐MB‐231 (B) at days 1, 2, 3, 4, and 7, together with the corresponding growth curves for MCF7 (C) and MDA‐MB‐231 (D). Scale bars: 200 µm. For panels C and D, each point represents one spheroid. An average of more than 370 spheroids was analyzed per seeding density and timepoint condition across two independent experiments performed using separate plates. The same wells were followed from day 1 to day 7. (E,F) Representative brightfield images of MCF7 (E) and MDA‐MB‐231 (F) spheroids embedded in collagen I inside the microfluidic chips at 2 and 24 h after loading. Scale bars: 200 µm. Whole‐chip views for each cell type are shown in Figure S1.

Importantly, the architecture was engineered to enable spheroid loading while preserving spheroid architecture during the injection process, favored by the expanded inlet geometry and the 1 mm channel height. The microfluidic design also incorporated sliding walls to precisely control the positioning and polymerization of the collagen gel containing spheroids, while enabling the central compartment to be opened or closed during medium exchange or NP loading. Once embedded in the collagen matrix of the microfluidic chips, MCF7 spheroids maintained a rounded, cohesive morphology at both 2 and 24 h post‐seeding (Figure 2E). Conversely, MDA‐MB‐231 spheroids exhibited an invasive pattern within 2 h of seeding, which intensified by 24 h, as cells progressively migrated into the adjacent collagen matrix (Figure 2F). Such behavior reflects the mesenchymal‐like characteristics of this cell line, including greater motility and invasiveness [35, 36].

Importantly, we adopted a prefabricated spheroid strategy using agarose microwells to ensure reproducible, size‐controlled spheroid formation for both BC cell‐line models, thereby providing standardized initial conditions and facilitating reliable integration into the ToC platform. Indeed, when evaluating self‐aggregation by seeding single cells directly into the chip, MCF7 cells formed only small, irregular aggregates, and MDA‐MB‐231 cells failed to form spheroids altogether. Representative images of these unsuccessful attempts are shown in Figure S2.

### 2.2 Responses to NP‐PTT in the Two Distinct BC Cell‐Line Models

AuNRs were chosen for NP‐PTT due to their strong absorption in the NIR spectrum and established photothermal properties. The optical properties of AuNRs, particularly their strong absorption in the NIR range, have been well‐documented in the literature [37, 38]. This absorption is critical for generating localized heat within the microfluidic device. In addition to their photothermal properties, polyethylene glycol (PEG)‐coated AuNRs were employed due to the well‐established stabilizing effect of PEGylation, which helps to reduce nonspecific surface interactions and improves colloidal stability [39].

Figure 3A provides an overview of the NP‐PTT workflow, in which spheroids were mixed with collagen and injected into the central channel of the chip, followed by incubation of AuNRs through the lateral channels prior to laser irradiation. The photothermal conditions were first evaluated in collagen‐filled chips without spheroids using three Au concentrations: 0.08, 0.4, and 2 mm (Figure 3B). Based on these preliminary tests, 0.4 mm was selected as the working Au concentration for subsequent experiments because it provided a measurable photothermal response while avoiding the highest NP‐loading condition. We next assessed three collagen concentrations (2, 4, and 6 mg.mL^−^^1^) as well as two incubation times, 2 and 24 h after NP administration (Figure 3C), with 4 mg.mL^−^^1^ used throughout the study because it better approximates breast tumor stiffness [40, 41]. Importantly, the microfluidic design incorporating sliding walls allows the central compartment to be opened during NP administration and subsequently closed during heating. AuNRs began diffusing into the collagen matrix within the first hours, producing a detectable but still submaximal temperature increase, with the largest measured temperature response observed after 24 h. Collagen concentration did not substantially affect the measured temperature response. Across all tested collagen concentrations, a similar trend was observed: approximately half of the maximum temperature increase was achieved within a few hours, while the largest measured response was reached at 24 h.

**FIGURE 3:**
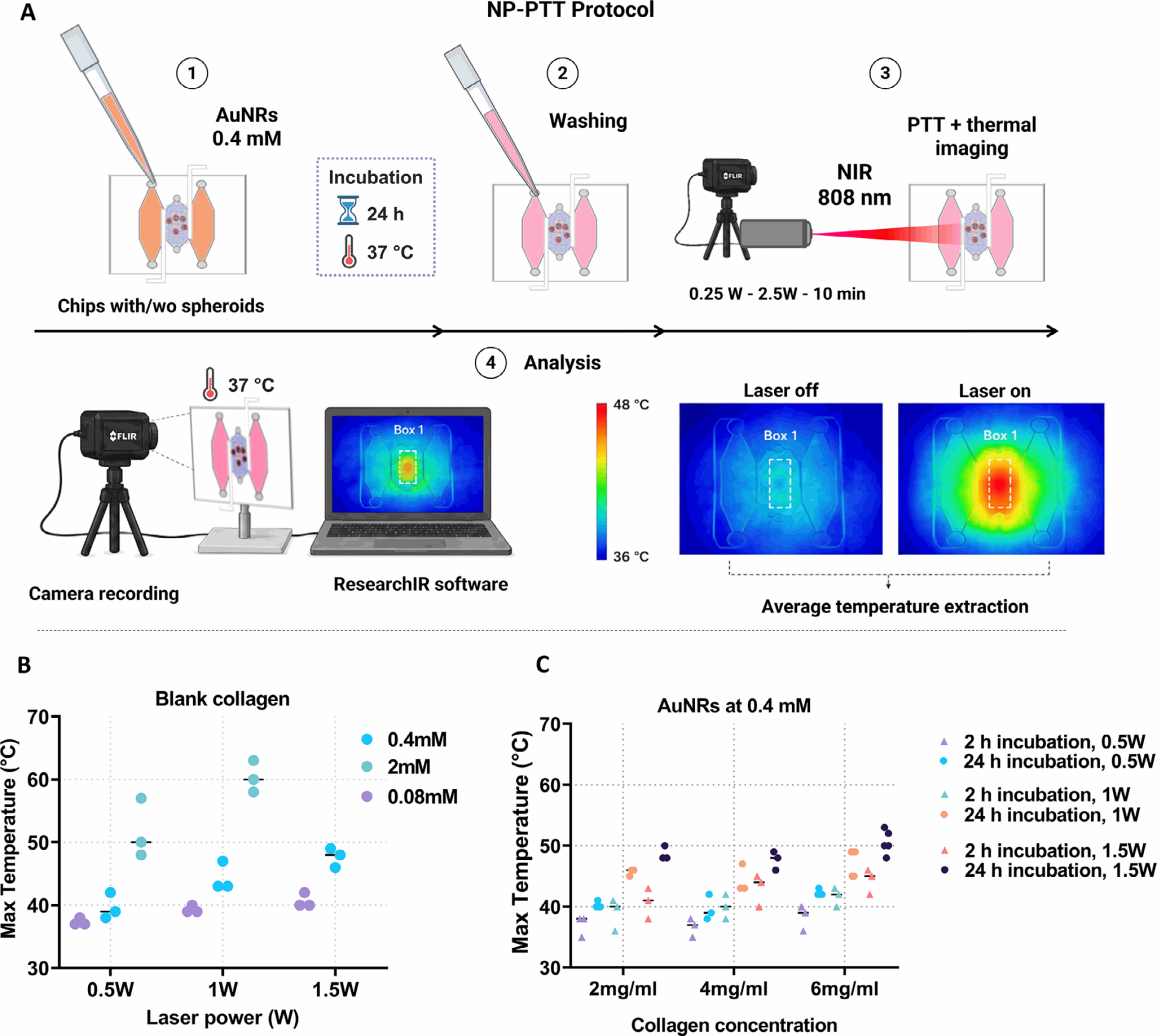
Schematic representation of the NP‐PTT protocol. (A) AuNRs were injected into the lateral channels of the microfluidic chip and incubated for 24 h. Afterward, the chips were washed and subsequently exposed to an 808‐nm NIR laser at different power settings for 10 min. A thermal camera was used to acquire images during the exposure, and the average temperature was later extracted using the ResearchIR software. Created with BioRender. (B) Maximum temperature reached in cell‐free collagen gels infused with AuNRs at different concentrations (0.08, 0.4, and 2 mm) and exposed to increasing laser powers (0.5, 1, and 1.5 W). (C) Photothermal response of collagen gels prepared at 2, 4, and 6 mg/mL, measured after 2 and 24 h of AuNR incubation (0.4 mm), at three laser powers (0.5, 1, and 1.5 W), showing the influence of collagen density, NP concentration, and incubation time on the thermal response. For panels B and C, individual points represent independent measurements (*n* = 3 per condition), and horizontal lines indicate medians.

After these preliminary optimizations, chips seeded with either MCF7 or MDA‐MB‐231 spheroids were incubated in the lateral channels with AuNRs at a concentration of 0.4 mm for 24 h. After incubation with AuNRs for 24 h, the sliding walls were closed, and the lateral channels were flushed with fresh medium to limit the presence of AuNRs outside the central collagen compartment. This configuration effectively confined AuNRs to the central region. Chips were then sealed with tape and placed in a sample holder connected to a water bath to maintain a stable temperature of 37°C, ensuring thermal stability throughout the PTT experiment. Subsequently, the microfluidic chips were irradiated with an 808 nm NIR laser for 10 min at six distinct power levels: 0.25, 0.5, 1.0, 1.5, 2.0, and 2.5 W, with the laser source positioned 6.1 cm above the chip surface. The average temperature increase was monitored across all experimental groups using a thermal camera.

For all chips containing either MCF7 or MDA‐MB‐231 spheroids, a clear power‐dependent temperature rise was observed. Importantly, the relationship between laser power and temperature increase followed an asymptotic trend for both cell types, as confirmed by nonlinear fitting analysis (*R^2^* = 0.9946 for MCF7 and *R^2^* = 0.9970 for MDA‐MB‐231). This indicates that the temperature approaches a plateau at higher laser power levels in both models (Figure 4A). Importantly, the heating curves were thus highly consistent between independent experimental replicates and across the two BC cell lines (Figure 4B,C), with closely overlapping temperatures, further underscoring the robustness of the methodology. This reproducibility reinforces the platform's utility for reliably correlating defined temperature thresholds with biological outcomes.

**FIGURE 4:**
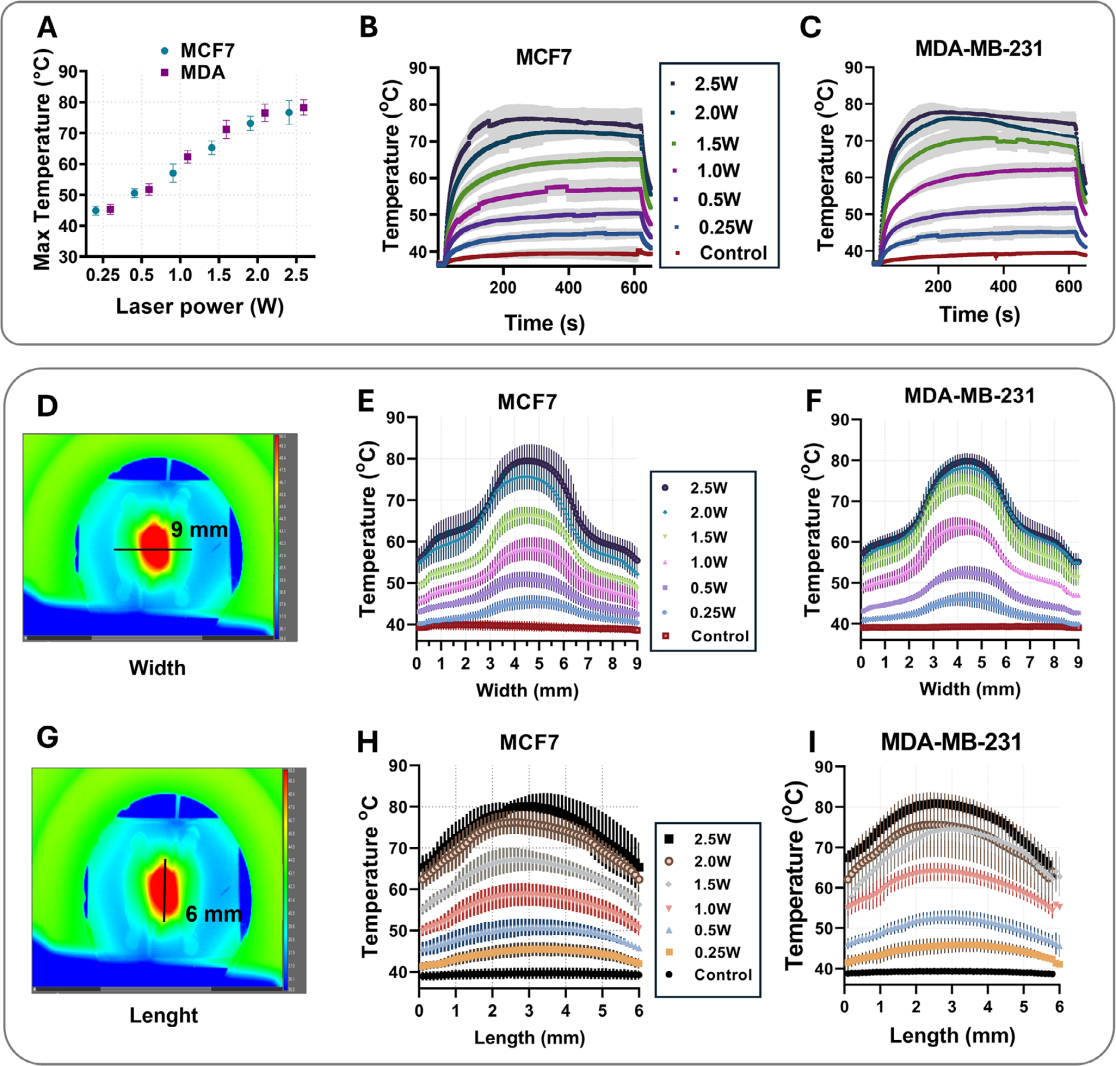
Thermal response and spatial temperature distribution during NP‐PTT in MCF7 and MDA‐MB‐231 spheroids. Data were obtained from four chips per condition across two independent experiments. (A) Maximum temperatures reached at each laser power for both cell lines. Data are presented as mean ± SD (standard deviation). (B,C) Temporal temperature profiles recorded over 10 min (600 s) of laser exposure for both MCF7 and MDA‐MB‐231 spheroids under six different laser power conditions (0.25–2.5 W), plus a control group exposed only to the laser at 2.5 W without AuNRs. Lines represent mean temperature values, and shaded areas indicate ± SD. (D) Temperature profile extracted across the chip width using ResearchIR software. (E,F) Spatial temperature distribution across the transverse section of the chip, including the central and lateral channels, under six laser power levels. (G) Temperature profile distribution extraction across the longitudinal section of the chip using the ResearchIR software. (H,I) Spatial temperature distribution across the chip length for the six laser power conditions. Temperature profile data are shown as mean values with shaded areas indicating ± SD.

Notably, spatial variations in temperature distribution were observed along both the transverse and longitudinal axes of the chips, with the highest temperatures confined to the central channel, which contained the collagen matrix and embedded spheroids. As illustrated in Figure 4D, temperature profiles were extracted along the transverse axis of the chip width using the ResearchIR software. The corresponding spatial heat maps (Figure 4E,F) show a marked temperature increase in the central channel, whereas heating in the lateral channels remained substantially less pronounced under all laser power conditions. Although the thermal profiles do not provide a direct quantitative measurement of AuNR confinement, these findings are consistent with localized AuNR‐mediated heating within the central region after lateral‐channel washing. Complementary transmission electron microscopy (TEM) analysis of AuNR‐exposed spheroids revealed electron‐dense structures consistent with AuNR‐containing intracellular vesicles in both MCF7 and MDA‐MB‐231 spheroids (Figure S3). As this analysis was performed on recovered spheroids, it provides qualitative evidence of AuNR‐cell interaction.

In contrast, longitudinal analysis (Figure 4G) involved tracing a profile along the length of the central channel at the laser focus site. The resulting temperature profiles (Figure 4H,I for MCF7 and MDA‐MB‐231 spheroids, respectively) revealed a modest but consistent increase in temperature along the channel length, with a clear peak at the laser irradiation site. As a control, chips irradiated in the absence of NPs showed no significant temperature increase, supporting that the measured thermal response under these irradiation conditions depended on the presence of AuNRs (black curves, Figure 4H,I).

Regarding the collagen I matrix, visible liquefaction was observed at temperatures above approximately 46°C in both cell‐line models, as shown in Figure 5A,B by the increased background brightness and loss of matrix structure. Among the tested laser powers, only the 0.25 W condition remained below this threshold, reaching approximately 45°C, whereas all conditions from 0.5 to 2.5 W exceeded 46°C. Although collagen thermal denaturation has been reported at temperatures around 55°C and above [42], matrix disruption was detected at lower temperatures in our system. This behavior may be attributed to differences in the experimental setting, heating dynamics, and the physicochemical environment [42]. In particular, the use of native, non‐crosslinked collagen at physiological pH and highly hydrated conditions may have increased the matrix's susceptibility to thermal remodeling [43], particularly under localized, sustained heating produced by NP‐PTT in the central collagen channel. More importantly, the chip's architecture (specifically the sliding wall design) enabled spheroids to remain confined within the central region even after collagen degradation. More importantly, the chip architecture, particularly the sliding‐wall design, maintained spheroids within the central compartment even after collagen degradation, allowing laser‐induced photothermal treatment to be completed while preserving the integrity of the experimental model.

**FIGURE 5:**
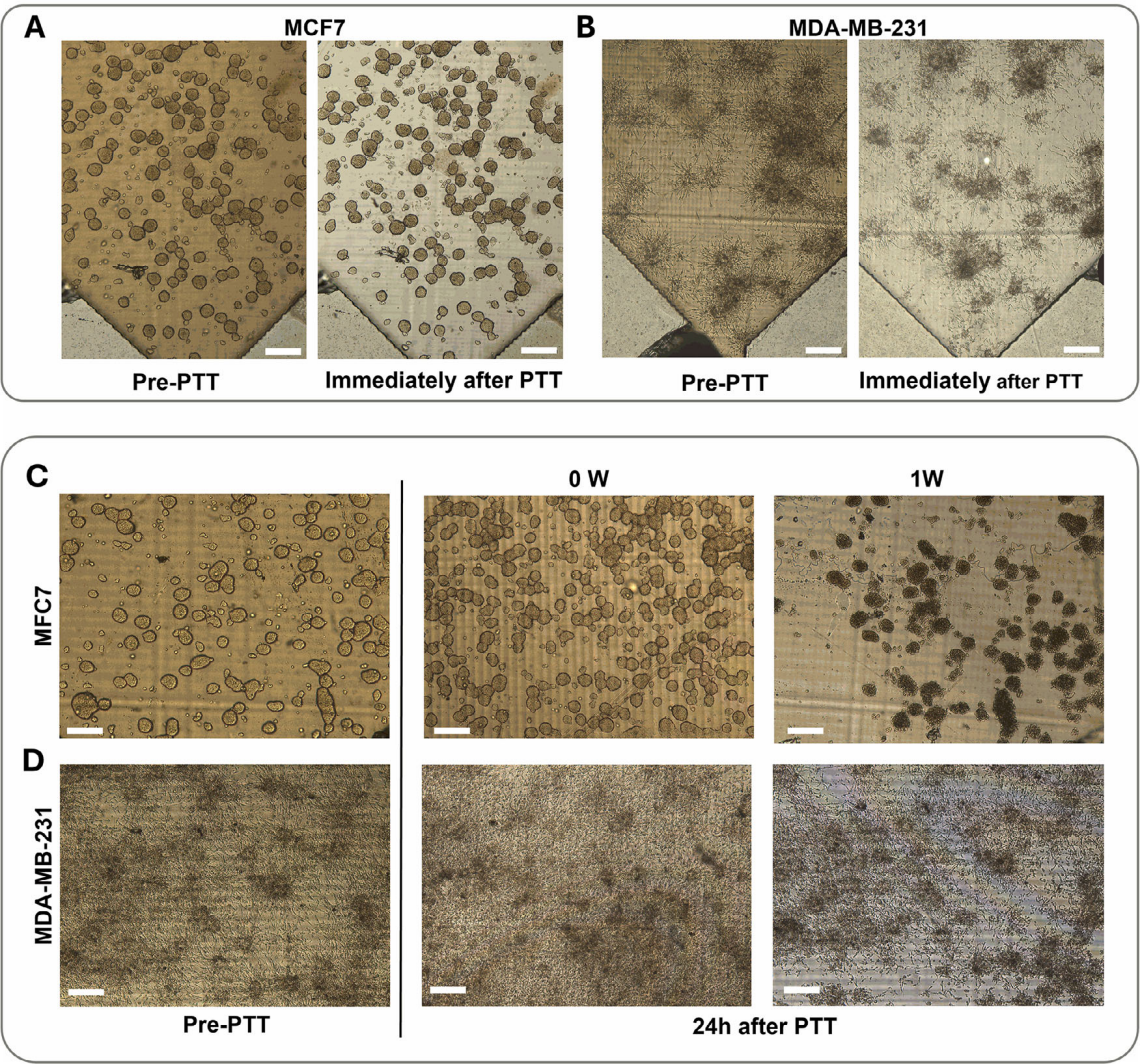
Morphological response of spheroids to NP‐PTT. Brightfield images of MCF7 (A) and MDA‐MB‐231 (B) spheroids, respectively, acquired before and immediately after PTT at 1 W. Representative images of MCF7 (C) and MDA‐MB‐231 (D) spheroid morphology and spatial organization before treatment and 24 h after NP‐PTT, compared with untreated controls maintained under the same experimental conditions without laser irradiation (0 W). Scale bar: 400 µm.

Figure 5C,D compares the morphology and spatial organization of MCF7 and MDA‐MB‐231 spheroids before treatment and 24 h after PTT treatment at 0 and 1 W. MCF7 spheroids before PTT appeared highly cohesive, rounded, and compact, with a well‐defined core, reflecting their epithelial phenotype. At 24 h after exposure, MCF7 spheroids retained their compact, well‐defined morphology in the absence of laser irradiation (0 W). Following PTT at 1 W, they largely preserved their overall structural organization but exhibited marked darkening, suggesting photothermal‐induced cellular damage and internal structural alterations, as previously reported for tumor spheroids exposed to cytotoxic treatments [44]. In contrast, before PTT, MDA‐MB‐231 spheroids exhibited a smaller, less compact core with irregular borders. Within a few hours after seeding, cells began to detach from the spheroid and invade the surrounding collagen matrix, resulting in a less cohesive morphology and more diffuse cellular distribution, consistent with the intrinsic mesenchymal and highly invasive phenotype of this cell line. Similar morphological features were observed in the 0 W control condition, indicating that laser‐free incubation did not markedly alter spheroid architecture. Following PTT at 1 W, MDA‐MB‐231 spheroids appeared more heterogeneous, with less defined spheroid boundaries and a more diffuse cellular distribution than the 0 W controls (Figure 5D).

### 2.3 Comparable Photothermal Cytotoxicity Across Distinct BC Cell Lines

To determine the cytotoxic effect of NP‐mediated PTT, both BC spheroid models were analyzed 24 h after treatment using live/dead fluorescence staining (Figure 6A,B). The experimental design included all six irradiation conditions, corresponding to laser powers of 0.25, 0.5, 1.0, 1.5, 2.0, and 2.5 W. Three control groups were evaluated in parallel: untreated spheroids, spheroids exposed to AuNRs in the absence of laser irradiation (0 W), and spheroids irradiated without AuNRs at the highest laser power (2.5 W). For each cell line and condition, four biological replicates were performed, using independent cell cultures and PTT sessions conducted on different days. Image‐based viability analysis was performed after spheroid segmentation. For each individual spheroid, Live and Dead fluorescence intensities were quantified independently, and the Live/Dead ratio was calculated. The resulting single‐spheroid data for MCF7 and MDA‐MB‐231 models are presented in Figure 6C,D, respectively, showing the distribution of Live, Dead, and Live/Dead fluorescence signals.

**FIGURE 6:**
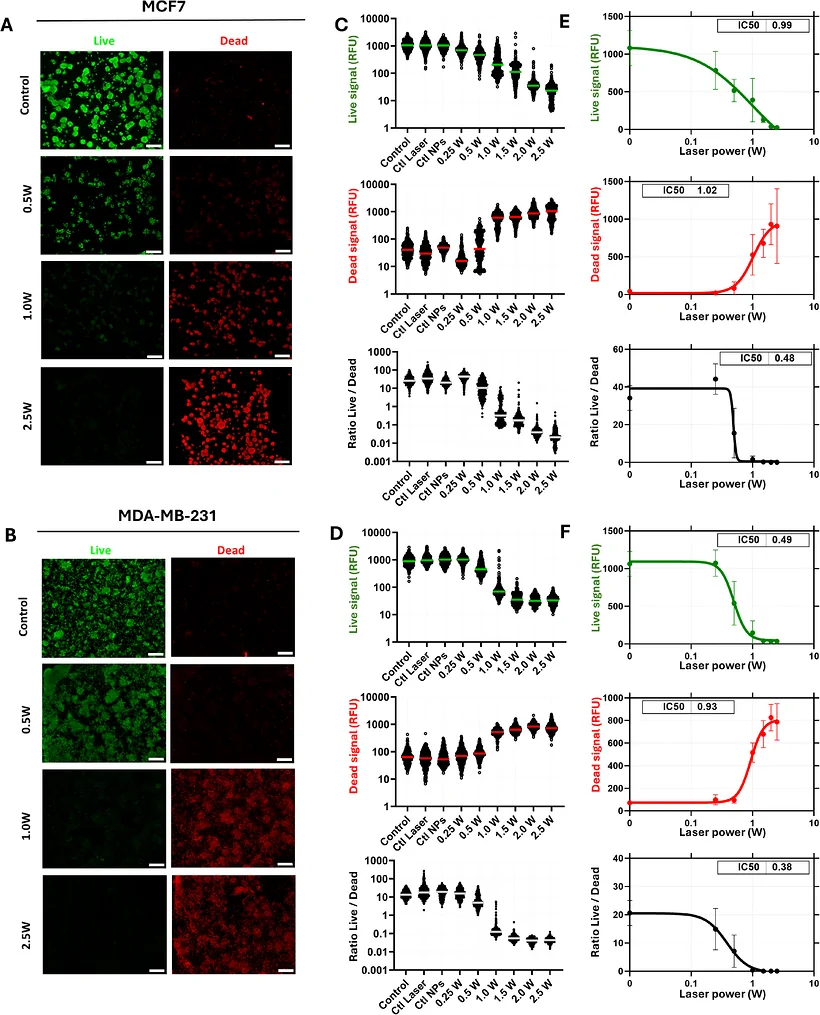
Cell viability analysis following NP‐PTT across varying laser power conditions. (A,B) Representative fluorescence microscopy images of MCF7 and MDA‐MB‐231 spheroids, respectively, for selected groups (Control, 0.5, 1.0, and 2.5 W), acquired 24 h after NP‐PTT. Live cells are shown in green, and dead cells in red. Scale bars: 500 µm. (C,D) Quantification of fluorescence intensity for live, dead, and live/dead ratios. Data were obtained from four chips per cell‐line model and treatment condition across two independent experiments performed on different days, with at least 200 spheroids analyzed per condition across the four chips. Bars represent the mean of the spheroid‐level measurements, and individual points represent spheroid‐level measurements pooled for visualization. (E,F) IC50 dose‐response curves fitted to fluorescence intensity data for live, dead, and live/dead ratios, plotted as a function of laser power. Control: no AuNRs, no laser; Ctl Laser: no AuNRs, laser 2.5 W; Ctl NPs: 4·10^10^ AuNRs/chip; laser 0 W.

Both cell lines exhibited a clear reduction in viability as the thermal dose increased. A consistent and pronounced decrease in viable cells was observed when the temperature exceeded approximately 50°C, corresponding to the 0.5 W irradiation condition, whereas lower laser powers produced only partial cytotoxicity. In contrast, the control conditions were characterized by intense Live fluorescence and minimal Dead signal, indicating that neither AuNR incubation nor laser exposure alone caused substantial cytotoxicity. Importantly, only the 0.25 W condition remained below the experimentally observed collagen‐liquefaction threshold (∼46°C), whereas all higher laser powers evaluated in the Live/Dead assay exceeded this threshold. Accordingly, collagen remodeling should be considered when interpreting cytotoxicity results obtained at powers ≥0.5 W.

This temperature‐dependent response is consistent with previous evidence indicating that cells may tolerate heating up to approximately 47°C via heat shock protein activation, thereby contributing to the maintenance of proteostasis under thermal stress [45]. Conversely, temperatures above 50°C have been associated with extensive cell death [45], in agreement with the effects observed in the present study. Despite the biological and phenotypic differences between MCF7 and MDA‐MB‐231 cells, the two spheroid models showed comparable sensitivity to NP‐mediated PTT under the tested conditions.

The treatment response was further quantified by calculating the half‐maximal inhibitory laser power (IC50). Live, Dead, and Live/Dead signals were summarized for each laser power and fitted using a standard IC50 model. The mean values were then plotted as a function of laser power and fitted using a standard IC50 model (Figure 6E,F). In MCF7 spheroids, the Live and Dead fluorescence curves converged at approximately 1.0 W. By contrast, MDA‐MB‐231 spheroids showed a more divergent profile, with values of 0.49 W for the Live signal and 0.93 W for the Dead signal. When cytotoxicity was assessed using the Live/Dead ratio, both cell lines showed closer responses, with ratio‐derived values of 0.48 W for MCF7 and 0.38 W for MDA‐MB‐231 spheroids. The similarity in the Live/Dead ratio‐based cytotoxicity response between MCF7 and MDA‐MB‐231 spheroids may be attributed, at least in part, to the thermal regime achieved during laser exposure. At most laser powers applied (≥1 W), temperatures measured in the spheroid‐containing central region exceeded 50°C, entering a heat‐ablation range in which proteins rapidly denature and cell membranes are disrupted [18]. According to Zhang and collaborators, cell death at these temperatures is predominantly associated with physical damage and necrotic mechanisms rather than with cell‐type‐specific metabolic or signaling pathways [46].

### 2.4 Assessment of Protein and Gene Expression Following NP‐PTT

To further investigate treatment‐induced changes in EMT‐associated markers, we performed immunofluorescence (IF) analysis 24 h post‐treatment (Figure 7A,B, additional images shown in Figures S4–S7), focusing on E‐cadherin, vimentin, and N‐cadherin expression, using at least 30 spheroids per condition (control, 0.5, and 1 W laser‐treated groups). Relative fluorescence intensity ratios (normalized to DAPI) and the vimentin‐to‐E‐cadherin ratio are shown in Figure 7C,D, with fluorescence measured across the entire spheroid for each channel. Ratios were calculated for each spheroid and averaged across replicates. As expected, MCF7 spheroids displayed a predominantly epithelial phenotype, whereas MDA‐MB‐231 spheroids exhibited a more mesenchymal phenotype, with a vimentin‐to‐E‐cadherin ratio almost three times higher in the latter. Following NP‐PTT, MDA‐MB‐231 spheroids showed no significant changes in the EMT‐associated markers analyzed. In contrast, MCF7 spheroids showed treatment‐associated modulation of selected epithelial‐ and mesenchymal‐associated proteins, with laser‐dose‐dependent increases in vimentin and N‐cadherin expression, a significant reduction in E‐cadherin, and an almost two‐fold increase in the vimentin‐to‐E‐cadherin ratio, approaching the value observed in MDA‐MB‐231 spheroids.

**FIGURE 7:**
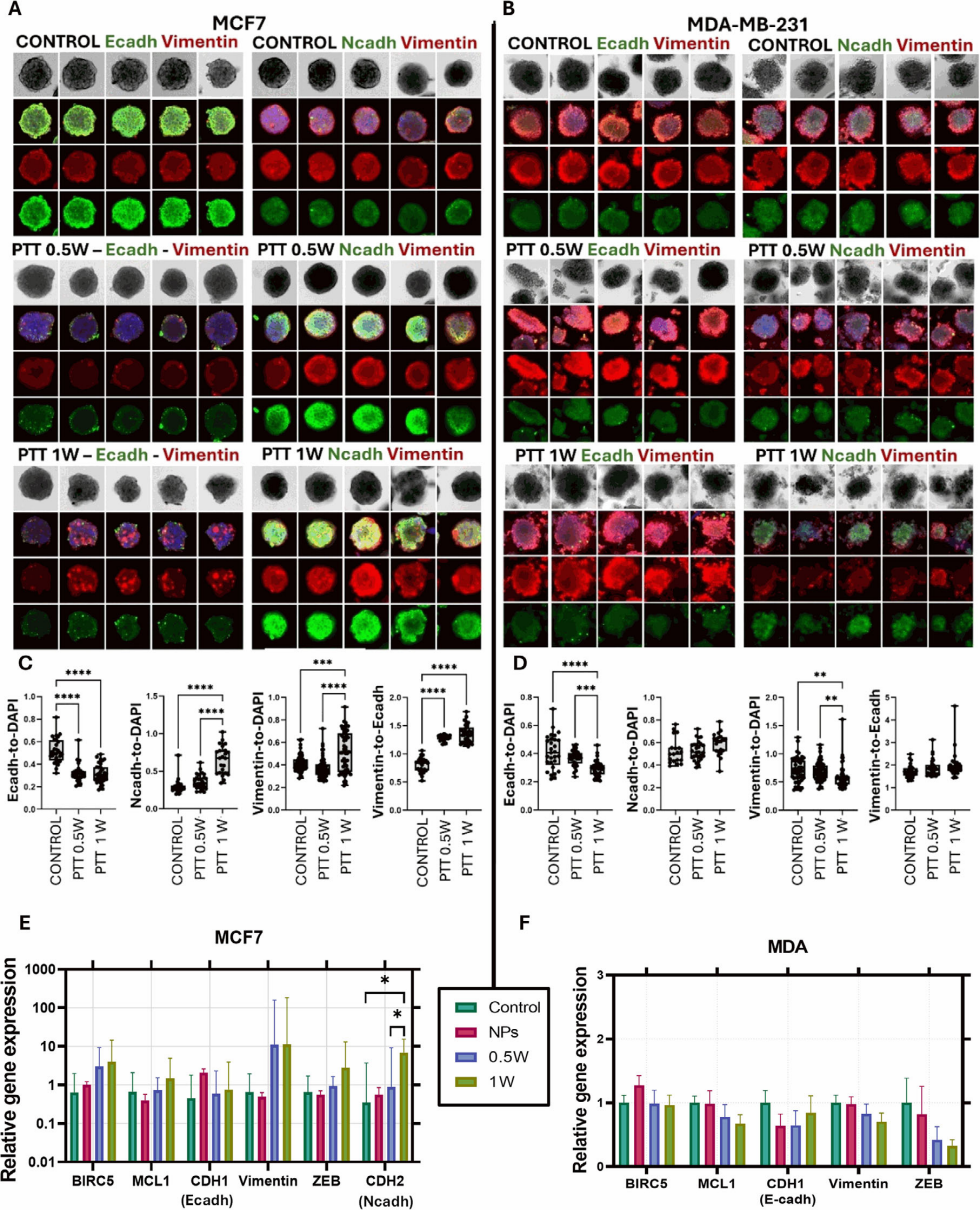
Immunofluorescence and gene expression analysis 24 h after NP‐PTT. (A,B) Representative Z‐projections of MCF7 and MDA‐MB‐231 spheroids immunostained for E‐cadherin (green), N‐cadherin (green), vimentin (red), and DAPI (blue). Images are shown for the untreated controls, 0.5, and 1.0 W laser‐treated groups. Each square measures 200 µm × 200 µm. (C,D) Quantification of E‐cadherin, N‐cadherin, and vimentin relative to the nuclei and the vimentin/E‐cadherin fluorescence intensity ratios. Signal intensities were measured across the entire spheroid area, and ratios were calculated per spheroid (≥30 spheroids per condition). Bars indicate group means and statistical significance was determined using one‐way ANOVA followed by Tukey's post hoc test, with ^*^ *p* ≤ 0.05, ^*^ *p* ≤ 0.01, *p* ≤ 0.001, and ^****^ *p* ≤ 0.0001. (E,F) Relative mRNA expression of apoptosis‐related genes (birc5 and mcl1) and EMT‐associated markers (cdh1, vim, cdh2, and zeb1) in MCF7 and MDA‐MB‐231 spheroids, respectively, following NP‐PTT. Treatment conditions include: Control, Control + AuNRs, 0.5, and 1.0 W. Data are presented as mean ± SEM (standard error of the mean) (*n* = 7 for MCF7, *n* = 3 for MDA‐MB‐231) where n represents independent pooled RNA samples, each generated by combining spheroids recovered from at least three chips under the same condition.

Next, we evaluated the expression of key genes involved in these pathways 24 h post‐treatment in both MCF7 and MDA‐MB‐231 spheroids. For apoptosis, we assessed the expression of baculoviral IAP repeat‐containing 5 (birc5), also known as *survivin*, and myeloid cell leukemia sequence 1 (mcl1), both of which are associated with survival signaling and therapy resistance, particularly in TNBC [47]. For EMT‐associated markers, we analyzed the expression of E‐cadherin (cdh1), vimentin (vim), and zinc finger E‐box‐binding homeobox 1 (zeb1), a transcription factor known to repress epithelial genes and to promote mesenchymal features [48], in both cell lines. In addition, N‐cadherin (cdh2) was assessed in MCF7 spheroids to further evaluate treatment‐associated modulation of mesenchymal‐associated markers in the initially epithelial‐like MCF7 model. The experimental groups included: non‐treated control group, NP‐only control after 24 h incubation, and PTT‐treated groups exposed to 0.5 and 1.0 W irradiation. Overall, quantitative polymerase chain reaction (qPCR) analysis revealed modest, gene‐specific transcriptional changes between the control and treated groups for both cell lines (Figure 7E,F), indicating that, at this timepoint, NP‐PTT induced limited gene‐level modulation in the surviving populations treated at 0.5 or 1.0 W. In MCF7 spheroids, a slight upregulation of BIRC5, the gene encoding survivin, was observed, likely reflecting a stress response to treatment. In contrast, MDA‐MB‐231 spheroids showed decreased mcl1 expression, consistent with a treatment‐associated transcriptional response in this TNBC model.

Regarding EMT‐associated genes, vim expression did not significantly change under laser‐treated conditions in MCF7 spheroids, whereas cdh2 expression was significantly upregulated following NP‐PTT. Indeed, the increased cdh2 transcript levels are supported by the enhanced N‐cadherin IF observed 24 h after treatment. Importantly, Lee et al. reported that sublethal hyperthermia (≤47°C) enhances mesenchymal markers [45]. Similarly, Dubrova et al. reported mild EMT characteristics in pancreatic cancer cells following magnetic NP‐mediated PTT, as evidenced by increased vimentin levels [49].

Photothermal damage was further assessed by hematoxylin and eosin (H&E) staining in spheroids from the control, 0.5, and 1 W groups (Figure 8A). The treated samples showed cellular damage throughout the spheroids under both laser exposure conditions, with no central necrosis observed, likely due to the relatively small spheroid size. Reverse transcription qPCR (RT‐qPCR) analysis of interleukin (IL)‐1β, IL‐6, and tumor necrosis factor (TNF)‐α revealed an upward trend in pro‐inflammatory cytokine expression following PTT, with a more pronounced increase at 1 W (Figure 8B). These results suggest that NP‐PTT may induce tumor cell‐intrinsic inflammatory or stress‐associated signaling, which could be further investigated in future studies incorporating immune components to evaluate tumor–immune interactions.

**FIGURE 8:**
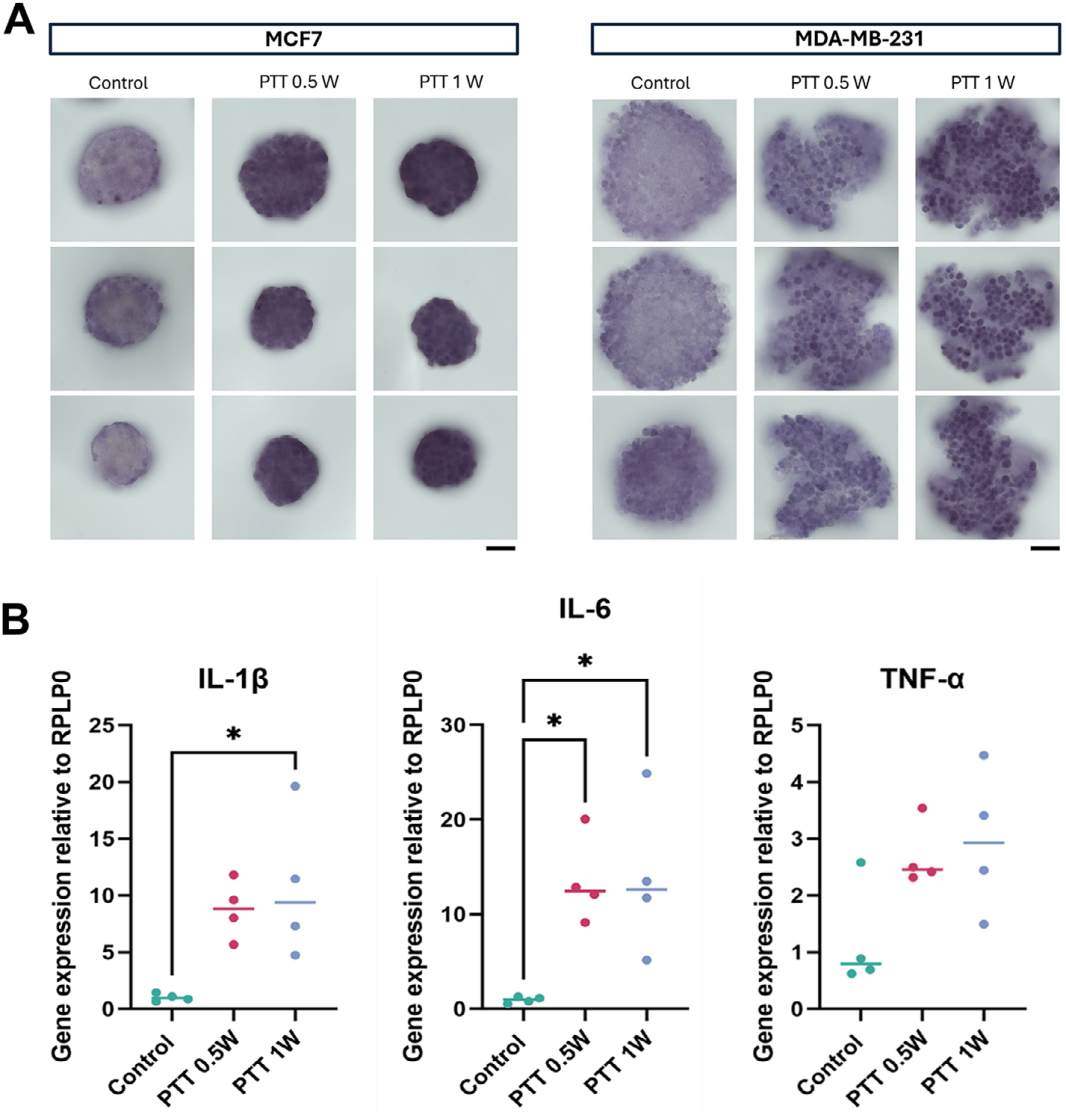
Histological analysis and inflammatory response after NP‐PTT. (A) Representative H&E‐stained sections of MCF7 and MDA‐MB‐231 spheroids recovered from the chips after NP‐PTT at 0.5 or 1 W, showing the spatial distribution of treatment‐induced cell damage. Scale bar = 50 µm. (B) Relative expression of pro‐inflammatory cytokines in MCF7 cells (IL‐1β, IL‐6, and TNF‐α) measured by RT‐qPCR in control chips and chips exposed to NP‐PTT at 0.5 or 1 W. Each point represents one independent pooled RNA sample generated by combining spheroids recovered from four or more chips under the same condition (*n* = 4 per condition). Expression values were normalized to RPLP0 and are shown relative to the control group. Horizontal lines indicate group means, and statistical significance was assessed using one‐way ANOVA followed by Tukey's post hoc test (*p* < 0.05).

## 3 Discussion

Cancer incidence continues to grow, despite significant efforts from researchers and healthcare providers [2]. Currently available cancer treatments, though effective, exhibit limitations, as not all tumor types respond equally, largely due to individual patient and tumor characteristics. Within this context, NP‐PTT emerges as a promising alternative, especially given its potential for precise tumor targeting and localized heat generation [50]. However, for NP‐PTT to advance toward broader preclinical and clinical relevance, a deeper understanding of how different tumor models respond under physiologically relevant conditions is necessary.

In this study, we aimed to improve the understanding of the NP‐PTT effects in a breast ToC model by investigating the thermal responses of two BC cell‐line models with distinct phenotypic and molecular backgrounds: luminal‐like MCF7 and TNBC MDA‐MB‐231 cells. We comparatively assessed spheroid formation, thermal sensitivity, gene expression, and EMT‐associated markers following NP‐PTT exposure within a controlled 3D microenvironment. The microfluidic device, fabricated from polydimethylsiloxane (PDMS) and equipped with a sliding wall mechanism, enabled collagen‐embedded spheroids to remain within the central channel even when matrix liquefaction was observed above approximately 46°C. This design maintained spheroid spatial positioning, enabling in situ analysis of cell behavior and treatment outcomes. Furthermore, the experimental design included AuNR‐only and laser‐only controls, allowing the biological effects of AuNR‐mediated photothermal therapy to be distinguished from those associated with NP exposure or laser irradiation alone. However, the absence of a temperature‐matched heat‐only (free hyperthermia) control precludes direct comparison between AuNR‐mediated photothermal treatment and equivalent NP‐free heating.

By using agarose microwells, we produced spheroids from each cell line before injecting them into the specially designed microfluidic chip. MCF7 spheroids, characterized by rapid, cohesive development, exhibited epithelial‐like features and robust intercellular adhesion. In contrast, MDA‐MB‐231 spheroids were more loosely packed and exhibited early spreading after embedding, consistent with their mesenchymal characteristics and dispersive behavior. These morphological and behavioral differences allowed us to explore how each cell‐line model responds to photothermal stress. A significant strength of this study is the choice to introduce prefabricated spheroids into the microfluidic device rather than relying on the self‐aggregation of individual cells. This approach is particularly beneficial when dealing with invasive cell lines such as MDA‐MB‐231, which generally struggle to form stable spheroids in situ. By creating spheroids externally in agarose microwells and subsequently placing them in a 3D collagen matrix, we facilitated more consistent spheroid size, structure, and positioning, even for highly motile cells.

The microfluidic platform's design, especially the central channel with sliding‐wall technology, enabled effective containment of the collagen‐spheroid matrix while allowing NP perfusion through the lateral channels. Closing the sliding walls and washing the lateral channels helped confine the AuNR‐containing collagen‐spheroid matrix to the central region, producing thermal profiles consistent with localized and reproducible heating within this compartment. Temperature measurements across varying laser power settings demonstrated similar thermal profiles for both cell lines, characterized by a nonlinear, plateau‐like increase in temperature with increasing laser power and closely aligned maximum temperature values. Both spheroid models also showed similar Live/Dead ratio‐based cytotoxic response patterns. Together, these findings support the ability of the ToC to provide controlled and reproducible thermal treatment conditions across two BC cell‐line models with distinct spheroid architectures.

IF analysis performed 24 h post‐treatment at 0.5 and 1 W provided additional insights into protein‐level changes in EMT‐associated markers induced by NP‐PTT, revealing increases with laser power in the vimentin/E‐cadherin ratio and N‐cadherin expression in MCF7 spheroids. These findings are consistent with modulation of selected mesenchymal‐associated markers following sublethal PTT, a response that has occasionally been reported after sublethal thermal stress [45, 49]. Beyond thermal and viability readouts, spheroids recovered after NP‐PTT were subjected to complementary histological and molecular analyses. H&E staining showed treatment‐associated cellular damage without an evident central necrotic region, although apoptosis and necrosis were not directly distinguished using mechanism‐specific assays. In parallel, RT‐qPCR analysis of recovered spheroids showed an upward trend in IL‐1β, IL‐6, and TNF‐α expression, particularly at 1 W. Together, these downstream analyses complement the thermal and viability assessments performed using the ToC and support its use as a treatment platform coupled to subsequent biological characterization.

Some limitations of the current study should be acknowledged. First, the 0.5 and 1 W conditions used for the IF and RT‐qPCR analyses exceeded the experimentally observed collagen‐liquefaction threshold. Therefore, the spheroids were exposed to NP‐PTT within a heat‐modified collagen microenvironment before recovery and downstream processing. The protein‐ and gene‐expression results should consequently be interpreted under these treatment conditions, as the independent contribution of collagen matrix alteration was not separately assessed.

Regarding AuNR characterization within the platform, TEM analysis provided qualitative evidence consistent with AuNR internalization by tumor cells, but NP uptake, dose, retention, and compartment‐specific spatial distribution within the chip were not quantitatively assessed. Moreover, the colloidal stability of the AuNR formulation under the specific culture medium and collagen conditions, photothermal conversion efficiency, and possible irradiation‐induced reshaping or changes in optical properties were not evaluated. Accordingly, the present study assessed the thermal response generated within the ToC platform rather than providing a complete physicochemical and photothermal characterization of the AuNRs. Additionally, the intrinsic dispersive behavior of the cells within the collagen matrix in the MDA‐MB‐231 model, together with the absence of well‐defined spheroid boundaries before treatment, hampered the reliable quantification of conventional invasion parameters, such as invasion distance and spheroid expansion. Finally, EMT‐ and survival‐associated responses were evaluated by RT‐qPCR and IF without complementary quantitative protein analyses, which should be considered when interpreting these molecular changes at the protein level.

Future studies may build on this platform by incorporating quantitative approaches to assess NP localization, cellular uptake, dose, retention, and compartment‐specific distribution, as well as AuNR colloidal stability, photothermal conversion efficiency, and potential irradiation‐induced reshaping or changes in optical properties. More detailed analyses of cell death mechanisms, together with complementary quantitative protein analyses such as Western blotting, could further strengthen the mechanistic characterization of NP‐PTT responses. The modular design of the ToC also enables the incorporation of additional cellular components, including normal epithelial, stromal, endothelial, and immune cells such as macrophages and lymphocytes, allowing the development of more complex TME models to support studies of more selective therapeutic strategies. Such models could also facilitate investigations of mild PTT (42°C–45°C), which has been proposed to reduce nonspecific thermal injury to normal tissues [51], as well as fractionated PTT, heat‐induced ECM remodeling, tumor–matrix and tumor–immune interactions, combinatorial therapies, drug screening, and patient‐specific therapeutic responses under increasingly complex 3D microenvironmental conditions.

Collectively, these findings position this ToC model as a robust and adaptable platform for investigating tumor responses to localized heating during NP‐PTT. Its compatibility with structural, molecular, and imaging analyses supports its use as a versatile 3D system for evaluating photothermal treatments and expanding toward more complex TME configurations.

## 4 Conclusion

This work presents a biomimetic ToC platform that reproduces key structural and functional features of the breast TME to investigate NP‐PTT. By integrating preformed spheroids into a perfusable collagen‐based microfluidic system, we achieved robust and reproducible generation of 3D tumor models while preserving distinct cell‐line model characteristics, including the dispersive phenotype of MDA‐MB‐231 cells.

Within this engineered microenvironment, the device enabled AuNR‐mediated heating and controlled assessment of photothermal responses. While temperatures above 50°C induced marked cytotoxicity in both BC models, sublethal photothermal treatment was associated, at 24 h post‐treatment, with increased protein expression of selected mesenchymal‐associated markers in MCF7 spheroids. Altogether, the platform provides a versatile breast ToC framework for integrated thermal, molecular, and phenotypic interrogation of NP‐PTT, indicating that responses to nanomedicine‐based hyperthermia may involve not only cytotoxicity but also increased expression of selected mesenchymal‐associated proteins following sublethal thermal stress in the epithelial‐like MCF7 model.

## 5 Experimental Section

### 5.1 Cell Culture

Human BC cell lines MCF7 and MDA‐MB‐231, commonly used as luminal‐like and TNBC models, respectively, were purchased from ATCC (American Type Culture Collection). Cells were maintained in Dulbecco's Modified Eagle Medium (DMEM; Gibco, UK) containing 10% fetal bovine serum and 1% penicillin‐streptomycin (Thermo Fisher Scientific, USA). Cultures were kept at 37°C in a humidified atmosphere with 5% CO_2_. When approximately 80% confluence was reached, cells were dissociated with 0.25% trypsin‐EDTA (ethylenediaminetetraacetic acid; Gibco, UK) and subsequently used to generate spheroids.

### 5.2 Spheroid Formation

Agarose‐based microwell molds were generated in conventional 6‐well plates using a 3D‐printed template containing micropillars measuring 200 µm in diameter and 200 µm in height. Briefly, each well was loaded with 2% agarose (A0576, Sigma–Aldrich, USA) prepared in phosphate‐buffered saline (PBS), and the template was then placed into the liquid agarose to imprint the microwell pattern. The agarose was left to gel at room temperature for 20 min, and the template was then gently detached, yielding approximately 2200 microwells in each mold. Before cell seeding, the agarose molds were sterilized by exposure to ultraviolet C (UVC) light for 1 h.

After sterilization, 0.5 million cells were seeded into each well of a 6‐well plate. The plates were then incubated under standard culture conditions to allow the cells to mature, aggregate, and form spheroids within the agarose molds before their use in the chip. Once formed, spheroids were gently harvested from the agarose molds, resuspended in culture medium, and transferred into a 15 mL conical tube. To separate spheroids from residual single cells, the tube was left undisturbed, allowing spheroids to sediment by gravity. The supernatant containing single cells was carefully removed, leaving a pellet of intact spheroids for further use. Only after this step were the spheroids mixed with collagen I (4 mg mL^−^^1^) and subsequently introduced into the ToC system.

### 5.3 Microfluidic Device Model

A custom microfluidic chip was developed to model a 3D TME. The device comprises a central gel channel (1 mm in height) for 3D culture and two lateral channels for the controlled injection of media, drugs, or NPs. This chip was specifically designed with a large inlet and a tall central channel to enable spheroid injection without compromising their integrity or causing clogging. Fabricated from PDMS, the chip also incorporates sliding wall technology [33], engineered to prevent spheroid escape from the central chamber in the event of collagen matrix degradation during NP‐PTT.

To fabricate the PDMS chip, a silicone elastomer base was mixed with curing agent (Sylgard 184, USA) in a 10:1 ratio and degassed to remove air bubbles. The molds were filled with the mixture and cured in an oven for at least 2.5 h. Following curing, holes were punched with 1.5 and 2 mm biopsy punches for the gel and media channels, respectively. The PDMS chips and glass coverslips were cleaned with isopropanol, dried, and plasma‐treated to ensure strong bonding.

For chip surface modification, dopamine hydrochloride was prepared in Tris‐HCl buffer at a final concentration of 0.2 mg mL^−^^1^. Approximately 25 µL was injected into the central channel of each device and left to react for 2 h at room temperature. After incubation, the channels were thoroughly rinsed with PBS, then dried and UVC‐sterilized to ensure aseptic conditions before further use.

### 5.4 Collagen Preparation and Spheroids Seeding in Microfluidic Chips

Rat tail‐derived type I collagen (Corning, USA) was prepared at a final concentration of 4 mg mL^−^^1^ by combining the collagen stock solution with 10× PBS, 1 N NaOH, and DMEM culture medium, adjusting the formulation to pH 7. To avoid premature gelation and preserve collagen properties, all preparation steps were carried out on ice. The spheroid pellet, comprising approximately 2000 spheroids, was subsequently resuspended carefully in the neutralized collagen solution.

Approximately 25 µL of the spheroid‐collagen solution was injected into the central channel of each microfluidic chip, using a dilution sufficient to introduce 300 to 500 spheroids per chip. The chips were incubated at 37°C with 5% CO_2_ for 30 min to allow complete collagen polymerization. Following gelation, DMEM was added to the lateral media channels, and the chip's sliding walls were opened to initiate perfusion of the media through the collagen matrix.

### 5.5 NP‐PTT Setup

PEGylated AuNRs were obtained from nanoComposix, USA (Figure S8). Additional in‐house analyses, including representative TEM images and a UV–vis–NIR spectrum, are shown in Figure S9 and are consistent with the manufacturer's information. Table 1 summarizes AuNR characterization.

**TABLE 1:** Summary of PEGylated AuNRs characterization. CV: Coefficient of variation.

Parameter	Reported value/observation
Morphology	Rod‐shaped NP
Mean length	49.9 nm
Mean width	12.2 nm
Length distribution	CV = 8.9%
Width distribution	CV = 7.5%
Aspect ratio	4.14
Hydrodynamic diameter	10 nm
Zeta potential	−38 mV
UV–vis–NIR absorption	λmax = 795 nm; OD800 = 50.7
Surface coating	PEG, mPEG 5 kDa

In addition, preliminary experiments were performed to compare PEG and citrate coatings, whose supplier characterizations (NanoComposix) are also presented in Figure S10. Citrate‐stabilized AuNRs showed less homogeneous diffusion within the microchannels, with a fraction of particles remaining attached to the channel walls, consistent with aggregation (Figure S11). PEG‐AuNRs were thus selected for the main study because they provided a more homogeneous distribution and a more controlled and reproducible photothermal response within the 3D microfluidic system.

The NPs were diluted in culture medium to a final gold concentration of 0.4 mm and perfused into the devices through the two lateral channels (25 µL per channel). Given the average dimensions of the synthesized AuNRs (49.9 × 12.2 nm), a 50 µL perfusion volume delivered approximately 4 · 10^10^ AuNRs per device. The chips were maintained at 37°C in a humidified 5% CO_2_ incubator for 24 h, allowing NP diffusion into the collagen‐spheroid construct located in the central channel. After this incubation period, the sliding walls were closed, and the lateral media channels were thoroughly rinsed with fresh culture medium to remove any non‐retained NPs. The devices were then sealed with PCR tape and transferred to a custom‐built holder coupled to a water bath, which maintained the system at approximately 37°C during irradiation.

NP‐PTT was performed using an 808 nm NIR laser for 10 min. Six irradiation conditions were evaluated as treatment groups, with four biological replicates per group (*n* = 4): 0.25, 0.5, 1.0, 1.5, 2.0, and 2.5 W. Experimental controls included untreated samples, AuNR‐only, and laser‐only conditions. A temperature‐matched heat‐only (free hyperthermia) control was not included. Therefore, the experimental design distinguished the effects of AuNR‐mediated PTT from those of AuNRs or laser irradiation alone, but not from equivalent NP‐free heating.

Over the 10 min irradiation period, the total delivered energy ranged from 150 to 1500 J. The laser was positioned 6.1 cm above the chip surface, based on a previously optimized configuration that enhances beam uniformity and minimizes spatial variation across the illuminated area [49], producing a laser spot of approximately 1 cm^2^ on the chip. Temperature changes during NP‐PTT were monitored using an infrared thermographic camera (A6751, FLIR). A region of interest (ROI) was selected along the central gel channel to extract average temperature values, which were exported as CSV files for further quantitative analysis.

### 5.6 Live/Dead Evaluation and Imaging Analysis

Spheroid viability was evaluated 24 h following NP‐mediated PTT using the LIVE/DEAD Cell Imaging Kit (R37601, Invitrogen, USA). The staining mixture was prepared according to the manufacturer's protocol, with the kit reagents diluted 1:2. For control samples and treatment conditions in which temperatures remained below 46°C, collagenase was used to digest the collagen matrix prior to staining. After the chip's sliding walls were sealed, the staining solution was perfused through the lateral media channels and incubated for 30 min at room temperature. Fluorescence images were then captured using a Nikon Eclipse Ti fluorescence microscope (Japan) coupled to a CCD camera and operated with a 2× objective.

Image quantification was carried out in ImageJ using 16‐bit grayscale images. Each fluorescence channel was analyzed independently following the same standardized procedure. Background subtraction was performed equally for the green channel, corresponding to Calcein‐AM‐positive viable cells, and the red channel, corresponding to BOBO‐3 iodide‐positive dead cells. ROIs were manually delineated over the central area of each spheroid and applied to both channels to obtain fluorescence intensity measurements. The green‐to‐red fluorescence ratios were calculated to estimate relative cell viability and subsequently used to derive IC50 values as a function of laser power for each cell line. Analysis included all NP‐PTT‐treated groups, as well as appropriate controls: untreated spheroids (Control), spheroids exposed to the highest laser power without NPs (Control Laser, 2.5 W), and spheroids treated with NPs alone (Control NPs, ∼4·10^10^ AuNRs/chip).

### 5.7 Relative Gene Expression by RT‐qPCR Analysis

Relative expression of apoptosis‐associated genes (birc5 and mcl1) and EMT‐related markers was evaluated 24 h after NP‐PTT. For EMT‐associated analysis, cdh1, vim, and zeb1 were assessed in both MCF7 and MDA‐MB‐231 spheroids, whereas cdh2 was evaluated only in MCF7 spheroids to further investigate treatment‐associated acquisition of mesenchymal features in the initially epithelial‐like model. As the reference gene for normalization, rplp0, encoding the ribosomal protein lateral stalk subunit P0, was used. Based on the IC50 values obtained in the cytotoxicity assays, four conditions were selected for RT‐qPCR analysis: Control, Control + NPs, 0.5 W, and 1 W. Total RNA was isolated using the NucleoSpin RNA extraction kit (Macherey‐Nagel, Germany), according to the manufacturer's protocol. Subsequently, 1 µg of RNA was reverse‐transcribed into cDNA using the High‐Capacity cDNA Reverse Transcription Kit (Thermo Fisher Scientific, USA). The primer sequences corresponding to each target gene are provided in Table 2.

**TABLE 2:** Primer sequences used for RT‐qPCR analysis in both cell lines.

Gene	Primer direction	Sequence (5′‐ 3′)
birc5	Forward	5′‐ AGC CAA ATT CGT TGT CAT AC‐3′
	Reverse	5′‐ GGT CTC CTC TGA CTT CAA CA‐3′
mcl1	Forward	5′‐ CAG CGA CGG CGT AAC AAA C‐3′
	Reverse	5′‐ACA AAC CCA TCC CAG CCT CTT T‐3′
cdh1	Forward	5′‐ ATT GCT CAC ATT TCC CAA CTC C‐3′
	Reverse	5′‐CTC TGT CAC CTT CAG CCA TCC T‐3′
vimentin	Forward	5′‐ TAC AGG AAG CTG CTG GAA GG‐3′
	Reverse	5′‐ACC AGA GGG AGT GAA TCC AG‐3′
zeb1	Forward	5′‐TTC ACA GTG GAG AGA AGC CA‐3′
	Reverse	5′‐ GCC TGG TGA TGC TGA AAG AG‐3′
cdh2	Forward	5′‐AGC CAA CCT TAA CTG AGG AGT‐3′
	Reverse	5′‐GGC AAG TTG ATT GGA GGG ATG‐3′

Amplification reactions were performed with SYBR Green PCR Master Mix (Thermo Fisher Scientific, USA) on a QuantStudio 3 Real‐Time PCR System (Thermo Fisher Scientific, USA). All samples were run in duplicate, and mean Ct values were considered for analysis. Expression levels were normalized to rplp0, and relative quantification was calculated according to the 2^−^ΔΔCt method [52]. For each condition, spheroids recovered from at least three chips processed within the same experiment were pooled to obtain sufficient material for RNA extraction. Each pooled RNA sample constituted one biological replicate.

### 5.8 Immunofluorescence Staining of Spheroids

IF staining was performed to assess E‐cadherin, N‐cadherin, and vimentin expression in MCF7 and MDA‐MB‐231 spheroids. Control spheroids were stained before insertion into the microfluidic devices, whereas treated samples were analyzed 24 h after NP‐mediated PTT performed at 0.5 or 1 W. Spheroids were recovered, fixed in 4% paraformaldehyde, and permeabilized with 0.1% Triton X‐100 in PBS for 20 min at room temperature. Blocking was then performed for 2–3 h at room temperature with PBS containing 2%–4% bovine serum albumin (BSA) and 0.1% Triton X‐100. For primary labeling, spheroids were maintained overnight at room temperature in blocking buffer supplemented with the following antibodies: mouse anti‐E‐cadherin monoclonal antibody (SHE78‐7, Invitrogen, USA; 1:200), mouse anti‐N‐cadherin antibody (aa. 802–819, BD Biosciences; 1:200), and guinea pig anti‐vimentin polyclonal antibody (GP53, ProGen; 1:100). Samples were subsequently washed three times in PBS for 30–45 min per wash. Overnight incubation with secondary antibodies was then performed in blocking buffer using Alexa Fluor 488‐conjugated goat anti‐mouse IgG (H+L) (Thermo Fisher Scientific, USA; 1:400) and Alexa Fluor 594‐conjugated donkey anti‐guinea pig IgG (1:100).

Following incubation with secondary antibodies, spheroids were rinsed three additional times in PBS, with each wash lasting at least 45 min. Nuclei were then counterstained with DAPI (Thermo Fisher Scientific, USA; 1:500 in blocking buffer), after which samples underwent three further PBS washes of at least 45 min each. Z‐stack images were acquired using a Leica DMi8 confocal microscope (Germany). Fluorescence quantification was performed in ImageJ using ≥30 spheroids per condition. E‐cadherin, N‐cadherin, and vimentin channels were analyzed independently, and signal intensities were extracted from 8‐bit images on a 0–255 scale. Marker fluorescence levels were normalized to the corresponding nuclear signal, and the vimentin/E‐cadherin fluorescence intensity ratio was calculated for each condition. Fluorescence intensities were measured across the entire spheroid area, and the vimentin/E‐cadherin ratio was calculated for each spheroid. At least 30 spheroids were analyzed per condition.

### 5.9 Histological and Molecular Analyses of Inflammatory Responses

H&E staining was performed following standard histological procedures to evaluate photothermal‐induced morphological alterations and cell death in MCF7 and MDA‐MB‐231 spheroids after laser irradiation at 0.5 and 1 W within the microfluidic chips. Following treatment, spheroids were recovered from the collagen matrix by collagenase digestion, fixed in 4% paraformaldehyde, embedded in paraffin, and sectioned. Tissue sections were subsequently stained with H&E and analyzed by optical microscopy.

Gene expression analysis of IL‐1β, IL‐6, and TNF‐α was performed by qRT‐PCR on MCF7 samples collected from microfluidic chips under three experimental conditions: untreated controls and chips subjected to photothermal therapy at laser powers of 0.5 or 1 W. Total RNA was extracted, reverse‐transcribed into cDNA, and amplified using gene‐specific primers. Relative gene expression levels were normalized to the housekeeping gene RPLP0 and expressed as fold change relative to the control group. Table 3 lists the primer sequences used for each target gene.

**TABLE 3:** Primer sequences used for RT‐qPCR analysis of cytokines.

Gene	Primer direction	Sequence (5′‐ 3′)
IL‐1β	Forward	5′‐ CCA CAG ACC TTC CAG GAG AAT G ‐3′
	Reverse	5′‐ GTG CAG TTC AGT GAT CGT ACA GG ‐3′
IL‐6	Forward	5′‐ CGG GAA CGA AAG AGA AGC TCT A ‐3′
	Reverse	5′‐ GGC GCT TGT GGA GAA GGA ‐3′
TNF‐α	Forward	5′‐ TCA ATC GGC CCG ACT ATC TC ‐3′
	Reverse	5′‐ CAG GGC AAT GAT CCC AAA GT ‐3′

### 5.10 Statistical Analysis

Data were initially tested for normality using the Shapiro–Wilk test. When applicable, statistical significance was assessed using one‐way ANOVA followed by Tukey's post hoc test for multiple comparisons, as specified in the corresponding figure captions. The exact sample size (n), experimental unit, and descriptive statistics used for data presentation are specified in the corresponding figure legends. Statistical analyses were conducted using GraphPad Prism software (version 8.2.1), and differences were considered statistically significant at *p* < 0.05.

## Funding

This work was supported by the European Union (ERC‐2019‐CoG project NanoBioMade 865629), Institut Pierre‐Gilles de Gennes (Investissements d'avenir ANR‐10‐EQPX‐34), ITMO PCSI HT on Chip no. 23CP060‐00, and Coordenação de Aperfeiçoamento de Pessoal de Nível Superior (CAPES, Finance Code 001).

## Declaration of the Use of AI

In preparing this manuscript, the authors used OpenAI's ChatGPT for language editing and text revision. OpenAI's ChatGPT was also used to generate three illustrative icons for Figure 3, based on the authors’ own original images, to adapt them to a scientific paper format. These elements were used only for visual illustration and do not constitute experimental data or results. All AI‐generated outputs were carefully reviewed for accuracy, and the authors take full responsibility for the content of the submitted manuscript.

## Conflicts of Interest

The authors declare no conflicts of interest.

## Supporting information




**Supporting File**: smll75343‐sup‐0001‐SuppMat.docx.

## Data Availability

The data that support the findings of this study are available from the corresponding author upon reasonable request.
