# Supplementary material for: A Biomimetic Breast Tumor‐On‐Chip Platform Reveals Mesenchymal Marker Upregulation Following Gold Nanorod Photothermal Therapy
Source: Small. 2026 Aug 24;22(60):e75343. doi: 10.1002/smll.75343 (PMC13711470; doi:10.1002/smll.75343)
Supplement: Supplementary file 1 [file SMLL-22-e75343-s001.docx]

**SUPPORTING INFORMATION**

**A biomimetic breast tumor-on-chip platform reveals mesenchymal marker upregulation following gold nanorod photothermal therapy**

Camila A. Salvego^1,2^, Hasti Honari^2^, Anastasiia Dubrova^2^, Stéphanie Descroix^2^, Martha S. Ribeiro^1*^, Aurore V. Walle^2*^, Claire Wilhelm^2*^

1. Centro de Lasers e Aplicações. Instituto de Pesquisas Energéticas e Nucleares (IPEN-CNEN), São Paulo, 05508-000, Brasil.
2. Laboratoire Physique des Cellules et Cancer, PCC, CNRS UMR168, Institut Curie, Sorbonne Université, PSL Research University, 75005 Paris, France

* marthasr@usp.br, aurore.van-de-walle@curie.fr, claire.wilhelm@cnrs.fr

**Figure S1.** Brightfield images of MCF7 (A) and MDA-MB-231 (B) spheroids embedded in collagen I inside the microfluidic chip 24 h post-loading. Scale bar: 200 µm.

**Figure S2. Representative images of MCF7 and MDA-MB-231 cells seeded directly into the microfluidic devices. A**) MCF7 single cells seeded at 5,000 cells/chip imaged on Days 0, 1, 3, and 8, forming only small and irregular aggregates. **B**) MDA-MB-231 cells seeded at the same density and imaged on the same days did not form spheroids. **C–D**) MDA-MB-231 cells were seeded at tenfold higher densities to assess whether increased cell numbers could promote spheroid formation. Scale bar: 200 µm.

**Figure S3:** TEM imaging of AuNRs internalized by MCF7 and MDA-MB-231 spheroids. Electron-dense AuNRs were observed inside endosomes in both spheroid models, indicating NP uptake by tumor cells. Scale bars: 10 μm and 2 μm, respectively.

**Figure S4.** Immunofluorescence analysis of MCF7 spheroids under control conditions and after laser irradiation at 0.5 W and 1 W. E-cadherin is shown in green, vimentin in red, and nuclei (DAPI) in blue. Each panel corresponds to a 200 × 200 µm field of view.

**Figure S5.** Immunofluorescence analysis of MCF7 spheroids under control conditions and after laser irradiation at 0.5 W and 1 W. N-cadherin is shown in green, vimentin in red, and nuclei (DAPI) in blue. Each panel corresponds to a 200 × 200 µm field of view.

**Figure S6.** Immunofluorescence analysis of MDA-MB-231 spheroids under control conditions and after laser irradiation at 0.5 W and 1 W. E-cadherin is shown in green, vimentin in red, and nuclei (DAPI) in blue. Each panel corresponds to a 200 × 200 µm field of view.

**Figure S7.** Immunofluorescence analysis of MDA-MB-231 spheroids under control conditions and after laser irradiation at 0.5 W and 1 W. N-cadherin is shown in green, vimentin in red, and nuclei (DAPI) in blue. Each panel corresponds to a 200 × 200 µm field of view.

**Figure S8. Manufacturer’s original data for PEG-AuNRs**

**Figure S9. TEM imaging (scale bar 500 nm) of the AuNRs and UV-Vis absorption spectrum.**

**Figure S10. Manufacturer’s original data for Citrate-AuNRs**

**Figure S11. Influence of surface chemistry on AuNR distribution and photothermal response:** (A) Maximum temperature at 0.6 W for the three tested AuNR concentrations (0.2, 0.4, and 1 mM), comparing PEG- and citrate-coated NPs. (B) Maximum temperature at 1 W under identical conditions. (C) and (D) Representative images of citrate-coated AuNRs at 1 mM within the microchannel, immediately after injection and 24 h postinjection, showing retention along the channel boundaries consistent with nanoparticle aggregation. Scale bar: 200 µm.
